# Low nicotine dependence and high self-efficacy can predict smoking cessation independent of the presence of chronic obstructive pulmonary disease: a three year follow up of a population-based study

**DOI:** 10.1186/s12971-015-0055-6

**Published:** 2015-08-28

**Authors:** Anne Lindberg, Benjamin Niska, Caroline Stridsman, Britt-Marie Eklund, Berne Eriksson, Linnea Hedman

**Affiliations:** Department of Public Health and Clinical Medicine, Division of Medicine, the OLIN unit, Umeå University, Umeå, Sweden; Department of Research, Norrbotten County Council, Luleå, Sweden; Department of Public Health and Clinical Medicine, Occupational and Environmental Medicine, the OLIN Unit, Umeå University, Umeå, Sweden; Krefting Research Centre, Institute of Medicine, Sahlgrenska Academy, University of Gothenburg, Gothenburg, Sweden

**Keywords:** Chronic obstructive pulmonary disease, COPD, Epidemiology, Nicotine dependence, Smoking, Smoking cessation

## Abstract

**Background:**

Smoking is a major risk factor for chronic obstructive pulmonary disease (COPD), and smoking cessation is the only intervention that slows disease progression. It is important to know whether current factors related to smoking and smoking cessation are different among subjects with and without COPD in order to support smoking cessation. The aim of this study was to evaluate factors related to smoking cessation and to compare characteristics and nicotine dependence among smokers with and without COPD.

**Methods:**

In 2005, 1614 subjects in a population-based longitudinal study of subjects with COPD and controls were examined. The Fagerström Test for Nicotine Dependence (FTND) and motivation for smoking cessation were assessed for current smokers (n = 299 total, 194 with COPD). Data on smoking cessation were collected in a follow-up in 2008 (n = 240).

**Results:**

Smokers with COPD had more pack-years and respiratory symptoms than smokers without COPD, whereas higher FTND scores were associated with anxiety/depression and respiratory symptoms in both groups. Nineteen percent of the smokers had quit smoking by the follow-up 3 years later, and they had significantly lower FTND scores (2.54 vs. 3.75, p < 0.001) and higher self-efficacy scores (10.0 vs. 6.0, p = 0.020) at baseline than the sustained smokers. Smoking cessation was related to low FTND scores and high self-efficacy independent of the presence of COPD, respiratory symptoms, anxiety/depression, and heart disease.

**Conclusions:**

The FTND score and a simple visual analog scale for assessing self-efficacy seem to be valuable instruments for predicting smoking cessation over several years, independent of COPD, respiratory symptoms, presence of anxiety/depression, and heart disease.

## Background

Chronic obstructive pulmonary disease (COPD) is estimated to become the fourth largest cause of death worldwide by 2030 [1, 2]. The most important risk factor for COPD is tobacco smoking [3], and every other elderly smoker eventually fulfills the spirometric criteria for COPD if they continue to smoke [4]. COPD is largely underdiagnosed, as less than one third of all cases are identified by health care providers [5]; thus, population-based studies are needed to obtain a true picture of COPD in the community. Cardiovascular diseases, the most common comorbidities in COPD, share the risk factor of smoking and contribute to the increased mortality and morbidity observed among subjects with COPD [6].

Smoking cessation is the most important intervention for preventing COPD and its progression [7] and for improving the prognosis of many comorbid conditions [8]. After quitting smoking, the accelerated decline in lung function slows down and nears that of non-smokers [9]. However, despite effective treatments [10], the nicotine in cigarettes is highly addictive and contributes to the difficulties smokers experience when trying to quit [11]. A few population-based studies in the late 1980s and early 1990s reported that smokers with COPD smoked more cigarettes per day and had higher nicotine dependence but had the same degree of motivation for smoking cessation as smokers without COPD [12, 13]. Awareness of nicotine dependence is important, as lower nicotine dependence has been shown to predict smoking cessation [14–16]. The proportion of smokers worldwide has gradually decreased, but the remaining smokers are more nicotine-dependent and may have more difficulty with quitting smoking [17]. It is, however, unclear if nicotine dependence and other factors related to smoking and smoking cessation differ between subjects with and without COPD in a society with low prevalence of smoking. Thus, an update of nicotine dependency and factors related to smoking cessation in relation to changing smoking behaviors are needed to provide relevant support for smoking cessation.

According to the Public Health Agency of Sweden, approximately 15 % of the Swedish population were smokers in 2005, the baseline of this population-based study. The aim of this study was to evaluate factors related to smoking cessation after 3 years, and to compare smokers with and without COPD regarding nicotine dependence, motivation for smoking cessation, respiratory symptoms, anxiety/depression, and heart disease.

## Methods

### Study design and study population

**Fig. 1:**
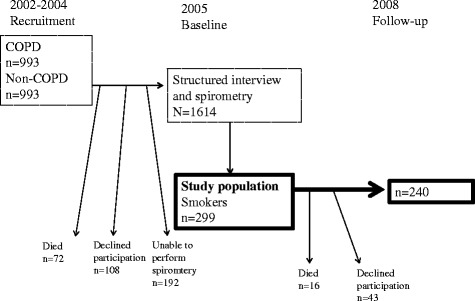
Flow chart of the study design and study population

The present study was performed within a population-based COPD study in the Obstructive Lung Disease in Northern Sweden (OLIN) studies. The study design has been presented in a previous publication [18]. Briefly, four large population-based cohorts were invited for re-examination including spirometry, in 2002–2004. We identified 993 subjects with COPD defined as FEV_1_/highest of FVC or VC <0.70, and 993 age- and sex matched subjects without obstructive lung function impairment (non-COPD) (Fig. 1). Since 2005, these subjects have been invited to annual examinations (i.e., once per calendar year) with a basic program including a structured interview and spirometry [18]. In 2005, 1614 individuals (81.3 % of the cohort) completed the interview and performed spirometry with acceptable quality.

The study population in the present study consisted of those who reported to be current smokers and had a complete score on the Fagerström Test for Nicotine Dependence (FTND) questionnaire (*n* = 299). A total of 240 subjects participated in the 3-year follow-up. The study was approved by the Regional Ethical Review Board at Umeå University, Sweden [Um dnr 04-045 M]. All participants provided informed consent to participate in the study.

### Questionnaires

The structured interview included questions about respiratory symptoms based on the Medical Research Council (MRC) questionnaire, including the modified MRC dyspnea scale (range 0–4) [19], and additional questions about smoking status, education, and comorbidities. Nicotine dependence was measured by the FTND questionnaire [20], a validated instrument assessing nicotine dependence on a scale from 0 to 10, with 10 being the greatest dependence. A questionnaire evaluating rapid engagement regarding motivation for smoking cessation included three questions derived from motivational interviewing [21]: “How motivated are you to quit smoking right now?” *(Motivation*), “How important is it for you to quit smoking now?” (*Importance*), and “How confident are you that you will succeed if you decide to quit?” (*Self-efficacy*). The answers were graded on a visual analogue scale from 0 to 10.

### Definitions

*Pack-years* was calculated using baseline data collected in 2005: number of cigarettes × years/20. Smoking habits were classified in 2008 as follows: *ex-smokers* had quit smoking more than 12 months ago*, continuous smokers* were current smokers or had quit less than 12 months ago. *Any respiratory symptoms* referred to a report of any of the following symptoms: chronic cough, chronic productive cough, recurrent wheezing, or dyspnea (defined as mMRC ≥2). *Anxiety/depression* was indicated by an affirmative response to the question “Have you had problems with worries, anxiety, or depression?” *Heart disease* referred to a report of any of the following: angina pectoris, past coronary artery bypass surgery, past percutaneous trans-luminal coronary angioplasty, past myocardial infarction, heart failure, or heart arrhythmia. Height and weight were measured and body mass index (BMI) calculated as weight in kilograms divided by height in meters squared, kg/m^2^). *Education* was divided into three categories: fulfilled compulsory school (primary and lower secondary school), gymnasium (upper secondary school), or higher education (college or university).

### Spirometry

Dynamic spirometry was performed using a dry volume spirometer (Dutch Mijnhardt Vicatest 5) according to American Thoracic Society recommendations. The best values from pre- or post-reversibility testing were used. COPD was defined as FEV_1_/highest of FVC or VC <0.70. COPD severity was based on FEV_1_ percent predicted according to the GOLD guidelines [1]: GOLD 1, FEV_1_ ≥ 80 % predicted, GOLD 2, 50 % ≤ FEV_1_ < 80 % predicted; GOLD 3, 30 ≤ FEV_1_ < 50 % predicted; and GOLD 4, FEV_1_ < 30 % predicted. The reference values from the Global Lung Function Initiative (GLI) [22] were used.

### Statistical analysis

The analyses were based on data collected in 2005 and information on smoking habits collected during the follow-up in 2008. Statistical analyses were performed using IBM SPSS statistics version 22. The cut-off value for significance was set to *p* < 0.05. Categorical variables were presented as percentages, and the chi^2^ test (or test for trend for variables with more than two categories) was used to detect significant differences. Normally distributed continuous variables (age, BMI, FTND-score) were presented as means with standard deviations (SD). For comparison of means the *T*-test was used, or ANOVA when there were more than two groups. Skewed variables were presented as medians with inter-quartile ranges (IQR) and the non-parametric median test used for comparisons between groups. The Pearson correlation test was used to detect significant linear associations for normally distributed variables, and the results were presented as correlation coefficients. Multivariate analyses were performed using multiple linear regression analysis for the linear variable FTND score and multiple logistic regression analysis for the categorical variable smoking cessation (i.e., fulfilling the definition of ex-smoker in 2008). Significant factors in the bivariate analysis were included as covariates in the multivariate analysis with sex, age, COPD, and pack-years.

## Results

### Baseline characteristics of the study population

The baseline characteristics of the 299 current smokers are shown in Table 1. Among COPD subjects, 44.8 % had GOLD 1, GOLD 2 51.5 %, and GOLD 3–4 3.6 %. Compared to non-COPD subjects, those with COPD were significantly older, had a lower BMI, and more pack-years. The prevalence of respiratory symptoms was significantly higher among subjects with COPD than non-COPD subjects, but there was no difference in the level of nicotine dependence, number of cigarettes smoked per day, motivation for smoking cessation, or prevalence of anxiety/depression or heart disease between groups.

**Table 1:** Baseline characteristics of the study population

		COPD	Non-COPD	p-value
		*n* = 194	*n* = 105	
Demographics				
	Female sex, n (%)	96 (49.5)	52 (49.5)	0.995
	Mean age, years (SD)	63.9 (9.6)	58.1 (9.1)	<0.001
	Mean BMI, kg/m^2^ (SD)	25.4 (4.0)	27.5 (4.9)	<0.001
Smoking				
	Pack-years, median (IQR)	23.8 (16.2-33.6)	18.0 (12.1-28.0)	<0.001
	Cigarettes/day, median (IQR)	10 (6–15)	10 (6–15)	0.981
	FTND score, mean (SD)	3.7 (1.8)	3.5 (2.1)	0.451
Motivation for smoking cessation, median (IQR)				
	Motivation (scale 0–10)	5 (4–8)	6 (5–8)	0.345
	Importance (scale 0–10)	8 (5–10)	8 (5–10)	0.464
	Self-efficacy (scale 0–10)	6 (3–10)	7 (5–10)	0.223
Education, n (%)				
	Higher education	23 (13.2)	14 (15.4)	
	Gymnasium	66 (37.9)	43 (47.3)	
	Compulsory	85 (48.9)	34 (37.4)	0.200
Comorbidities, n (%)				
	Any respiratory symptoms	180 (92.8)	78 (74.3)	<0.001
	Anxiety/depression	39 (20.1)	12 (11.4)	0.057
	Heart disease	34 (17.5)	15 (14.3)	0.470

*IQR* inter-quartile range

### Factors related to higher nicotine dependence

No significant differences were found in mean FTND scores between COPD and non-COPD subjects by sex, education, respiratory symptoms, anxiety/depression, or heart disease (Table 2). Anxiety/depression was associated with higher nicotine dependence in both COPD and non-COPD subjects, whereas respiratory symptoms were associated with higher nicotine dependence among non-COPD subjects. No significant difference was found when comparing FTND score within the GOLD stages: 3.7, 3.6 and 3.3 for GOLD 1, 2, and 3–4, respectively (*p* = 0.803). In both COPD and non-COPD subjects, there was a significant correlation between a higher number of pack-years and a higher FTND score, whereas higher self-efficacy was inversely correlated with the FTND score (Table 3). In COPD subjects, the FTND score was also negatively correlated with age.

**Table 2:** Mean Fagerström Test for Nicotine Dependence (FTND) scores at baseline (2005)

		COPD (*n* = 194)	p-value^a^	Non-COPD (*n* = 105)	p-value^a^	p-value^b^
Sex						
	Female	3.4 (1.8)		3.2 (2.1)		0.461
	Male	3.9 (1.9)	0.074	3.8 (2.1)	0.153	0.734
Education						
	Higher education	3.7 (2.0)		3.4 (2.3)		0.713
	Gymnasium	3.7 (1.8)		3.5 (2.3)		0.737
	Compulsory	3.7 (1.9)	0.998	3.7 (1.9)	0.876	0.867
Respiratory symptoms						
	No	3.0 (1.9)		2.5 (2.1)		0.375
	Yes	3.7 (1.8)	0.083	3.9 (2.0)	0.002	0.547
Anxiety/depression						
	No	3.4 (1.7)		3.3 (2.1)		0.482
	Yes	4.5 (2.0)	0.003	5.2 (1.6)	0.001	0.325
Heart disease						
	No	3.7 (1.8)		3.4 (2.2)		0.252
	Yes	3.6 (2.0)	0.738	4.1 (2.0)	0.202	0.364

Data are given as mean and standard deviation (SD)

^a^Within groups: female vs. male, etc

^b^COPD vs. non-COPD

**Table 3:** Correlation between Fagerström Test for Nicotine Dependence (FTND) scores and various factors at baseline (2005)

		COPD		Non-COPD	
		r	p-value	r	p-value
	Age × FTND	(−0.188)	0.009	(−0.149)	0.129
	Pack-year × FTND	0.304	<0.001	0.342	<0.001
Motivation for smoking cessation					
	Motivation × FTND	(−0.041)	0.571	(−0.110)	0.268
	Importance × FTND	0.006	0.936	(−0.161)	0.103
	Self-efficacy × FTND	(−0.281)	<0.001	(−0.267)	0.006

In a multivariate linear regression analysis, respiratory symptoms, anxiety/depression, and pack-years were significantly associated with higher FTND scores, whereas age and *s*elf-efficacy were negatively associated with the FTND score. In this model, COPD was not significantly associated with the FTND score (Table 4).

**Table 4:** Factors associated with Fagerström Test for Nicotine Dependence (FTND) score in multivariate linear regression analysis at baseline (2005)

	p-value	β	95 % CI
Male sex^a^	0.094	0.34	−0.06- 0.74
Age^b^	<0.001	−0.05	−0.07- -0.03
COPD^c^	0.727	0.08	−0.36- 0.52
Respiratory symptoms^d^	0.038	0.58	0.33- 1.13
Anxiety/depression^e^	<0.001	1.19	0.66- 1.72
Pack-years^b^	<0.001	0.03	0.02- 0.05
Self-efficacy^b^	<0.001	−0.13	−0.19- -0.07
Constant	<0.001	5.82	4.38- 7.26

ANOVA F-test p < 0.001 R^2^ = 0.269 R^2^ adjusted = 0.251

*β* beta-coefficient, *CI* confidence interval

^a^Reference group: female sex

^b^Continuous variable

^c^Reference group: non-COPD

^d^Reference group: no respiratory symptoms

^e^Reference group: no anxiety/depression

### Participation at follow-up

Among the 299 subjects who participated in the baseline examinations in 2005, 240 (80.3 %) participated in the follow-up 3 years later in 2008. Of the 59 individuals who did not participate in the follow-up, 16 had died. The baseline data did not differ significantly for the 59 subjects lost to follow-up and the 240 who participated in the 3-year follow-up regarding the presence of COPD, respiratory symptoms, nicotine dependence, pack-years, or the motivation for smoking cessation, but those who did not participate were older and had significantly more heart disease (data not shown).

### Factors related to smoking cessation

**Fig. 2:**
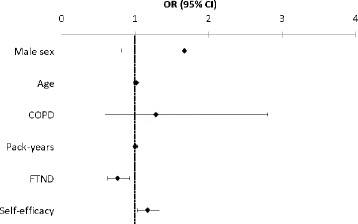
Factors related to smoking cessation, analyzed by multiple logistic regression analysis and expressed as odds ratios (OR) with 95 % confidence interval (CI). FTND: Fagerström test for nicotine dependence

The smoking cessation rate, i.e., the prevalence of non-smokers, among those who participated in the follow-up in 2008 was 19.2 % (*n* = 46). Nine participants were classified as continuous smokers. Among those who had stopped smoking, none had participated in a smoking cessation program, 21 % had used nicotine replacement therapy, 13 % had used snus, and 63 % reported quitting without any specific support. In bivariate analysis, no significant differences were found in sex, age, education level, respiratory symptoms, anxiety/depression, heart disease, or pack-years (data not shown) between ex-smokers and continuous smokers. However, quitters had significantly lower FTND scores (2.54 vs. 3.75, *p* < 0.001) and higher self-efficacy scores (10.0 vs. 6.0, p = 0.020) at baseline than those who had continued to smoke. In a multivariate analysis, smoking cessation was significantly associated with low FTND scores (OR 0.76, 95 % CI 0.63-0.93) and high self-efficacy scores (OR 1.17, 95 % CI 1.03-1.33) at baseline and after adjusting for the covariates sex, age, COPD, and pack-years (Fig. 2). When each of the variables heart disease and anxiety/depression were added to the model, none of them reached significance, and the previous significant relationships did not change.

## Discussion

This population-based study was performed in Sweden when approximately 15 % of the general population were smokers. Even though the smokers with COPD had more pack-years and respiratory symptoms than those without COPD, both groups had similar levels of nicotine dependence and motivation for smoking cessation. Anxiety/depression and respiratory symptoms were associated with higher nicotine dependence in both groups. Low nicotine dependency and high self-efficacy to quit smoking were each significantly associated with smoking cessation, which was defined as stopping smoking at least 12 months prior to the 3-year follow-up, and these factors remained significantly associated with smoking cessation after adjusting for age, sex, pack years, COPD, and the presence of heart disease or anxiety/depression.

In contrast to other population-based studies, which have demonstrated higher nicotine dependence among smokers with COPD [12, 13, 23], our study showed similar nicotine dependence levels irrespective of COPD status even though those with COPD had a higher burden of smoking as assessed by pack-years. Furthermore, those with respiratory symptoms had greater nicotine dependence than those without respiratory symptoms. A review suggested that smokers in countries with a lower prevalence of smoking have higher nicotine dependency [17]. This supports a hypothesis that decreased smoking prevalence results in difficulties helping the remaining smokers quit smoking even though they have developed respiratory health problems. However, the FTND scores observed in our study are relatively low in relation to the prevalence of smokers in society compared to the review [17]; all of the studies in the review with a smoking prevalence ≤ 20 % had FTND-scores >4. The reason for this difference is unclear, but our study included very few heavy smokers, which is important when calculating the FTND score. Maybe the Swedish smoking legislation during the last decade that prohibit smoking in restaurants and other public places has contributed to less smoking among smokers.

Anxiety/depression is a common comorbidity in COPD [24] and in this study, more common among smokers with COPD than smokers without COPD. Smokers with anxiety/depression had greater nicotine dependence than those without anxiety/depression, but nicotine dependence was similar among COPD and non-COPD. Major depression [25] and anxiety [26] have previously been linked to nicotine dependence, and our study shows that it may be important to recognize mental malaise among both subjects with COPD and those without COPD when providing support for smoking cessation. However, further studies are needed to evaluate whether specific interventions can improve smoking cessation support in this group [27].

Every fifth smoker had quit by the 3-year follow-up. The only intervention to support smoking cessation was simple advice to quit smoking at the end of the structured interview at the annual examination. Only a few comparable studies have evaluated long-term smoking cessation over several years. In a review published in 1995, the natural quit rate without interventions after 12 months varied between 13.8 and 8.5 %, and in a meta-analysis the rate of smoking cessation was 7.3 % during a mean follow-up of 10 months. However, it was not clearly defined how long he participants had to be smoke-free to be classified as having quit [28]. In a large international cohort study following >21,000 smokers for 5 years, 15.5 % had quit successfully, i.e., had been smoke-free for at least 12 months [29]. In a recently published study of treatment-seeking smokers followed for 52 weeks, the abstinence rate was 20.3 % among subjects randomized to nicotine replacement therapy compared to 23.8 % in a control group [30]. Treatment-seeking individuals can be expected to be more motivated than the average subject in our population-based study. Thus, the limited intervention in our study seems to have yielded a rather high proportion of quitters at the 3-year follow-up, especially in a society with an already rather low smoking prevalence. Furthermore, the majority of those who had stopped smoking had not participated in smoking cessation programs. The relatively low FTND scores may have facilitated smoking cessation, but the annual lung function testing may also have increased subjects’ understanding of the relationship between smoking and respiratory disease and contributed to the decision to quit smoking. These results imply that brief advice in connection with spirometry may increase the motivation to quit smoking, and these are simple methods that can easily be implemented in health care. Another contributor to the smoking cessation rate during the observation time may be the changes in legislation for stricter tobacco control; a law was passed in 2005 that banned smoking in all restaurants in Sweden.

Greater self-efficacy, i.e., confidence in one’s own ability to quit smoking when deciding to do so, was also related to smoking cessation in this study. In a review published in 2009, self-efficacy was associated with higher successful smoking cessation, but the relationship between self-efficacy and smoking cessation could be overestimated if the analysis does not adjust for smoking status at the time of assessment [31]. Self-efficacy is often assessed by multiple item questionnaires. However, our study of current smokers confirmed that self-efficacy defined by merely one question and a simple visual analog scale can predict smoking cessation within 3 years. Given the simplicity, this question could be a useful tool in general clinical practice and at smoking cessation clinics to identify smokers with an increased ability to quit smoking and further individualize support in relation to the expected outcome.

In smokers with smoking-related conditions, such as heart disease or COPD, smoking cessation is highly motivated. However, neither heart disease nor COPD or respiratory symptoms were associated with smoking cessation. Thus, a smoking-related condition itself does not provide enough motivation for smokers to quit. A difference in nicotine dependence among subjects with or without these conditions was also not an explanatory factor. Even though the correlation between smoking and various conditions is an obvious and integral part of the information given in smoking cessation support, it is probably not enough to motivate smoking cessation. Support for self-efficacy and the handling of high nicotine dependence seem to be important for smoking cessation. The observed positive correlation between pack-years and nicotine dependence as well as negative correlation between self-efficacy and nicotine dependence are reasonable to expect among smokers, however, with the reservation that a significant correlation does not ensure biological relevance.

The strength of this study is that the smokers were derived from a large population-based COPD cohort and controls without COPD, providing a representative picture of the average smoker with and without COPD. The under-diagnosis of COPD is substantial [5], and a population-based study of COPD such as our study would include a majority of mild to moderate cases of COPD, whereas registry-based studies would include mainly moderate to severe and very severe COPD cases. Well-validated methods, such as standardized spirometry, questionnaires for structured interviews, and the assessment of nicotine dependency by the FTND score contribute to high internal and external validity. However, data on comorbidities were collected by structured interview and not confirmed by medical records; thus, recall biases and misclassifications cannot be excluded. However, the risk of false positive results is small, as recall bias and misclassifications are likely to be randomly, and not systemically, distributed. Furthermore, smoking habits were not confirmed by objective measurements, such as cotinine levels. The spirometric criteria for defining COPD can also be discussed. A weakness of the fixed ratio is that it may underdiagnose COPD among younger people and overdiagnose COPD among the elderly. However, the current study was designed when the fixed ratio, FEV_1_/FVC < 0.70, was recommended to define COPD after the launch of the GOLD guidelines. If a similar study was designed today, the lower limit of normal (LLN) would be a better option according to current recommendations for defining COPD in epidemiological studies [32]. The fixed ratio is still commonly used in health care, and our results can be interpreted in relation to clinical practice, even though the weaknesses of the fixed ratio always have to be taken into account.

The analysis of non-participation indicates apotential ‘healthy smoker effect’, as subjects participating in 2005 but not 2008 were older, had significantly more heart disease, and close to one-third of them had died. This ‘healthy survivor effect’ has to be taken into account when interpreting data in epidemiological studies.

## Conclusion

In this population-based study, nicotine dependence assessed by the FTND questionnaire and motivation for smoking cessation were similar among smokers with COPD and those without COPD. Anxiety/depression and respiratory symptoms were associated with higher nicotine dependence in both groups of subjects. The study was performed in Sweden, a country with a relatively low prevalence of smokers. Similar to studies in countries with a higher prevalence of smokers, the most important factors associated with smoking cessation in a 3-year follow-up were low nicotine dependence and high self-efficacy, independent of the presence of COPD, heart disease, or anxiety/depression. Thus, the FTND score and a simple visual analog scale for assessing self-efficacy seem to be valuable instruments for predicting smoking cessation over several years in a society with a relatively low prevalence of smoking.
